# Kleine-Levin Syndrome: a diagnosis not to be forgotten in young adults

**DOI:** 10.1192/j.eurpsy.2026.11529

**Published:** 2026-07-31

**Authors:** C. Freitas, M. Felizardo

**Affiliations:** Unidade Local de Saúde de Matosinhos, Matosinhos, Portugal

## Abstract

**Introduction:**

Kleine–Levin syndrome (KLS) is a rare recurrent hypersomnia disorder characterized by episodes of excessive sleep, cognitive disturbances, hyperphagia, and often marked behavioral disinhibition or hypersexuality. The etiology remains unclear, though dysfunction of hypothalamic and thalamic circuits and autoimmune mechanisms have been proposed. Due to its fluctuating course and psychiatric-like manifestations, KLS is frequently misdiagnosed as a primary mood, psychotic, or behavioral disorder.

**Objectives:**

To reflect on the Kleine-Levin diagnosis and on the characteristics that hinder diagnosis and delay appropriate treatment.

**Methods:**

To describe a case report and to discuss the difficulties in the diagnosis, specifically in young adults.

**Results:**

A 22-year-old male was referred to psychiatric services for escalating hypersexual behavior, irritability, and periods of social withdrawal over the past year. Initially, the symptoms were attributed to an impulse control disorder, and psychotherapy was initiated without improvement. Over time, his family observed recurrent episodes lasting 7–10 days in which the patient slept up to 18 hours per day, exhibited voracious appetite, disorientation, and inappropriate sexual advances, followed by complete recovery with no recollection of the events. Between episodes, he functioned normally and denied depressive or psychotic symptoms. Neurological examination was normal. Laboratory tests, thyroid function, and toxicology screening were unremarkable. Brain MRI and EEG excluded structural or epileptiform abnormalities. Polysomnography revealed prolonged total sleep time during episodes without REM abnormalities. Based on clinical criteria and exclusion of alternative causes, a diagnosis of Kleine–Levin syndrome was established. Symptomatic treatment with modafinil during episodes provided mild benefit, while psychoeducation and structured sleep monitoring helped reduce anxiety and prevent risk behaviors.

**Conclusions:**

KLS typically affects adolescent males and follows a relapsing–remitting course, with episode frequency decreasing over time. The hypersexual and disinhibited behaviors observed in this case often lead to misinterpretation as mania, substance misuse, or personality disorder. Neuroimaging studies suggest transient thalamic hypoperfusion during symptomatic periods. Early recognition is crucial to prevent unnecessary psychiatric interventions and social stigmatization.

This case underscores the diagnostic complexity of Kleine–Levin syndrome, particularly when hypersexuality dominates the clinical presentation. Comprehensive neurological and psychiatric evaluation, along with exclusion of metabolic or epileptic disorders, is essential for accurate diagnosis. Increased awareness of KLS among clinicians may lead to earlier identification, more appropriate management, and improved quality of life for affected individuals.

**Disclosure of Interest:**

None Declared

